# Endoscopic Ultrasound-Guided Gastrojejunostomy for Superior Mesenteric Artery Syndrome Secondary to Rapid Weight Loss

**DOI:** 10.14309/crj.0000000000000868

**Published:** 2022-10-06

**Authors:** Andrew C. Storm, Tala Mahmoud, Karl Akiki, Ryan J. Law

**Affiliations:** 1 Mayo Clinic, Rochester, MN

## Abstract

A 19-year-old man diagnosed with diffuse large B-cell lymphoma undergoing chemotherapy presented for recurrent emesis and weight loss. Imaging studies of the abdomen demonstrated features of superior mesenteric artery syndrome. The patient deferred conservative treatment options and was deemed not to be a surgical candidate. Endoscopic ultrasound-guided gastroenterostomy using a lumen-apposing metal stent was performed to bypass the obstruction. Subsequently, the patient's oral intake and weight significantly improved. The stent was removed 6 months after placement with resolution of superior mesenteric artery syndrome symptoms.

## INTRODUCTION

The lumen-apposing metal stent (LAMS) was first approved for drainage of walled-off pancreatic necrosis in 2013.^1^ Since then, its off-label use has expanded to include endoscopic ultrasound-guided gastroenterostomy (EUS-GE), endoscopic ultrasound-guided biliary drainage, and endoscopic ultrasound-directed transgastric endoscopic retrograde cholangiopancreatography.^2^ EUS-GE is a minimally invasive procedure for the treatment of gastric outlet obstruction. This is performed by obtaining access to the jejunum through the stomach through the deployment of a LAMS.^3^ EUS-GE has been previously described in the literature for the management of SMA syndrome.^4–7^ We report the application of EUS-GE in the management of superior mesenteric artery (SMA) syndrome secondary to rapid weight loss in a young patient.

## CASE REPORT

A 19-year-old man presented with symptoms of shortness of breath and syncope associated with significant and rapid weight loss of 26.2% total body weight loss within 3 months. Imaging of the chest showed a large mediastinal mass, along with pericardial effusion and pneumothorax. Tissue biopsy confirmed the diagnosis of diffuse large B-cell lymphoma, and chemotherapy was initiated.

**Figure 1.**
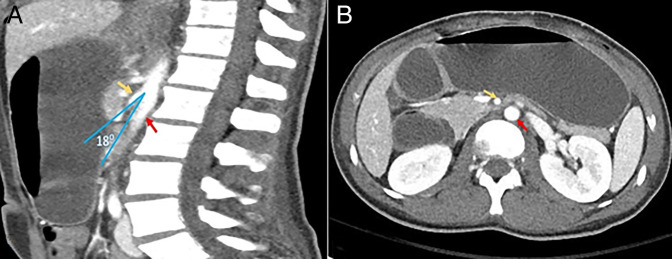
(A) Abdominal computed tomography sagittal view: Aortomesenteric angle = 18°; (B) dilatation of the stomach and the first part and second part of the duodenum, which transitions into decompressed bowel loops beyond the SMA. Yellow arrow = SMA; red arrow = aorta. SMA, superior mesenteric artery.

During hospitalization, the patient had recurrent emesis and was unable to tolerate oral intake. He continued to lose weight, reaching a nadir of 46.1 kg. An abdominal computed tomography (CT) scan with contrast revealed features consistent with SMA syndrome resulting in benign gastric outlet obstruction (GOO) from this extrinsic compression (Figure 1).

Treatment options were explored and included placement of a nasojejunal feeding tube in addition to a venting nasogastric tube, followed by future placement of a percutaneous gastrostomy with a jejunal feeding extension tube. However, the patient declined these options. Moreover, he was deemed not to be fit for surgery because of considerable risk of wound nonhealing secondary to ongoing chemotherapy. Therefore, the decision to proceed with EUS-GE was made.

**Figure 2.**
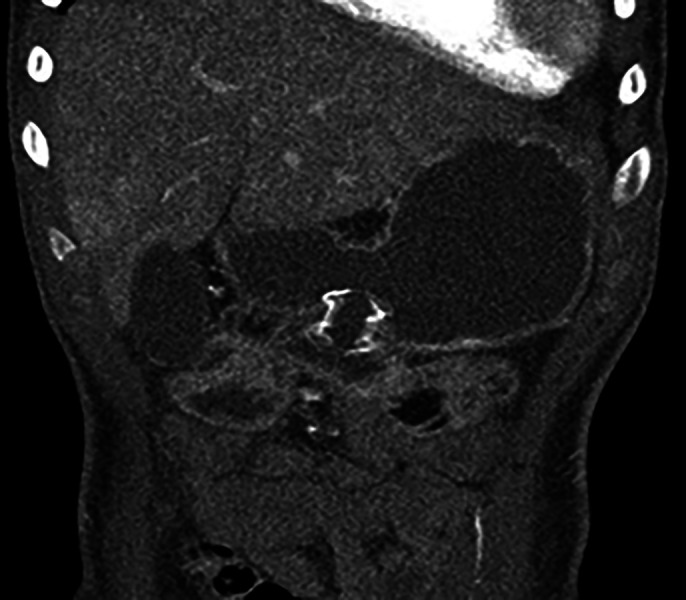
Abdominal computed tomography scan showing the gastrojejunal lumen-apposing metal stent.

Under a general anesthetic, the patient was placed in a partial left lateral position. A 10-French nasobiliary drain was placed over a guidewire to the proximal jejunum. Dilute contrast was injected through this drain beyond the area of duodenal obstruction to create a target for 12EUS access. A total of 4 mg of intravenous glucagon was given to slow small bowel motility. Using a freehand technique, a 15 × 10 mm electrocautery-enhanced LAMS was deployed across the stomach and distal duodenal lumens to create a gastroenterostomy effectively bypassing the obstruction (Figure 2). Within 24 hours, the patient was tolerating a full liquid diet and was discharged 9 days later on a low-residue mechanical soft diet.

One month later, the patient developed epigastric and right upper quadrant pain. A hepatobiliary iminodiacetic acid scan demonstrating bile reflux into the stomach ruled out cholecystitis, and the patient was treated conservatively with ursodiol. Follow-up upper gastrointestinal (GI) series during the same visit showed a widely patent gastroenterostomy.

**Figure 3.**
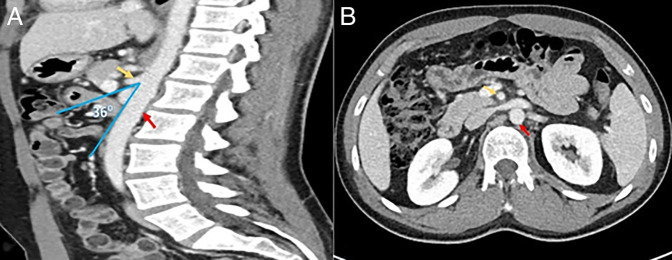
(A) Aortomesenteric angle 36°; (B) resolution of duodenal compression with a wider distance between the aorta and SMA. Yellow arrow = SMA; red arrow = aorta. SMA, superior mesenteric artery.

**Figure 4.**
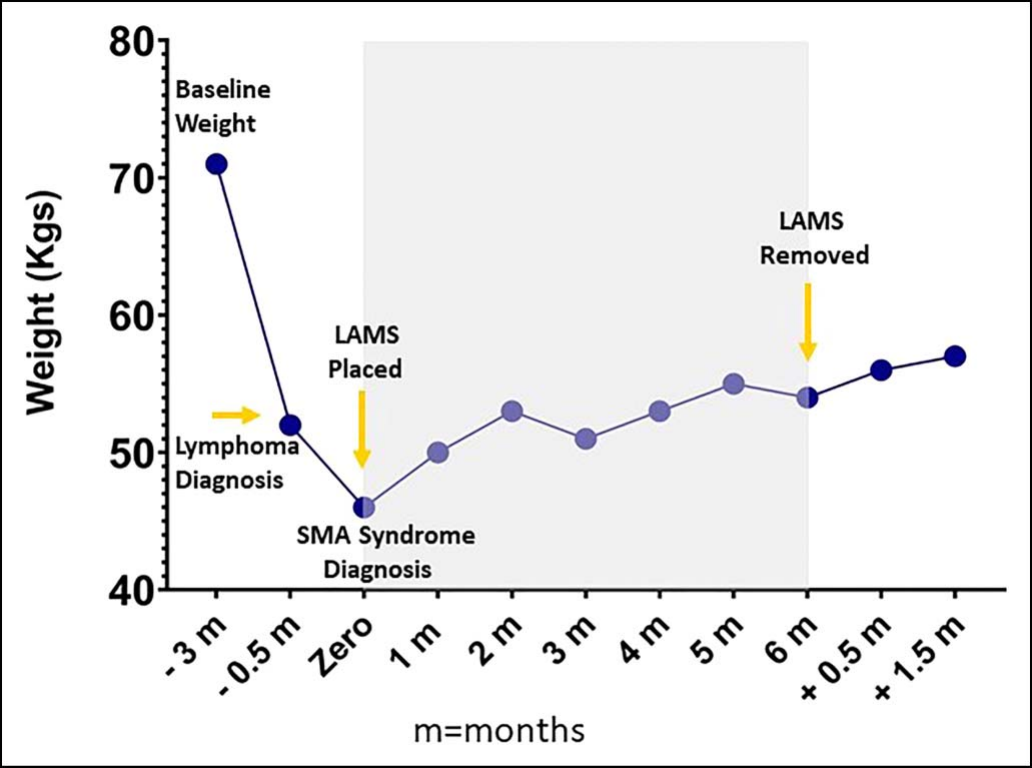
Weight trends according to SMA syndrome progression and resolution. LAMS, lumen-apposing metal stent; SMA, superior mesenteric artery.

Four months after LAMS placement, an upper GI series showed a patent gastroenterostomy. A follow-up abdominal CT scan (Figure 3) was also performed at that time and showed no compression at the third part of the duodenum. Six months after LAMS placement, the patient gained sufficient weight reaching a weight of 54.9 kg (Figure 4).

Given the earlier findings of the CT scan and upper GI series, the LAMS was removed. On the 6-week follow-up after removal, the patient continued to gain weight reaching 57.3 kg and was tolerating a regular diet.

## DISCUSSION

SMA syndrome is a rare cause of GOO and occurs secondary to reduction in the angle between the superior mesenteric artery and the aorta. This is usually caused by severe medical conditions resulting in rapid weight loss that leads to thinning of the mesenteric fat pad.^8^ The patient presented in this report had substantial weight loss secondary to lymphoma B symptoms exacerbated by chemotherapy and prolonged hospitalization in the intensive care unit setting. The clinical scenario along with CT scan findings led to a diagnosis of SMA syndrome.

The normal aortomesenteric angle ranges between 25° and 60°, and an angle of less than 22° is considered one of the diagnostic criteria of SMA syndrome in the proper clinical settings.^9^ In this case, improvement of the aortomesenteric angle from 18° to 36° and resolution of SMA syndrome were observed after weight regain.

Xu et al described the first case of SMA syndrome management with a reverse endoscopic ultrasound-guided technique. The patient reported 4.5-kg weight gain after 2 months of LAMS placement.^5^ Kouanda et al reported a case of an 80-year-old man with SMA syndrome who was also treated with EUS-GE. However, the patient had recurrence of SMA syndrome 3 months after LAMS removal and, thus, required yearly replacement of LAMS.^4^ Our patient experienced a net weight gain of 8.8 kg at 6 months after EUS-GE and maintained a stable weight at 6 weeks after LAMS removal suggesting that this technique may be a one-and-done procedure for select patients, particularly when the cause of weight loss (treatable malignancy in this case) is reversible.

EUS-GE is a safe and efficacious procedure in the management of malignant GOO,^10^ also demonstrating a high clinical success rate in patients with benign GOO.^11^ A similar efficacy and improved adverse event profile has been reported for EUS-GE among patients with malignant GOO when compared with surgical gastrojejunostomy.^12^ Although further study is needed, safety and efficacy of EUS-GE in the management of SMA syndrome has been reported in earlier case reports^4–7^ and was further demonstrated by our case.

EUS-GE provides a minimally invasive transoral endoscopic alternative to surgical gastroenterostomy. Where technical expertise exists, EUS-GE may be considered as a therapeutic option for patients with SMA syndrome as an alternative to surgical duodenal bypass, particularly for patients who are not fit for surgery. Future studies will help define the routine role of EUS-GE for patients with GOO from a variety of malignant and nonmalignant etiologies.

## DISCLOSURES

AC Storm is the article guarantor.

M. Alqaisieh, T. Mahmoud, and K. Akiki report no disclosures. RJ Law is a consultant for ConMed and Medtronic. AC Storm is a consultant for Apollo Endosurgery, ERBE, GI Dynamics, and Olympus and received research grant support from Apollo Endosurgery, Boston Scientific, Endo-TAGSS, Endogenex, and Enterasense.

All authors met ICMJE criteria for authorship rights.

Informed consent was obtained for this case report.

This case was not presented before at any meetings.

## References

[1] Bhenswala P Lakhana M Gress FG Andalib I. Novel uses of lumen-apposing metal stents: A review of the literature. J Clin Gastroenterol 2021;55(8):641–51. 34049379 10.1097/MCG.0000000000001566

[2] Mussetto A Fugazza A Fuccio L Triossi O Repici A Anderloni A. Current uses and outcomes of lumen-apposing metal stents. Ann Gastroenterol 2018;31(5):535–40. 30174389 10.20524/aog.2018.0287 PMC6102456

[3] Dawod E Nieto JM. Endoscopic ultrasound guided gastrojejunostomy. Transl Gastroenterol Hepatol 2018;3:93. 30603729 10.21037/tgh.2018.11.03 PMC6286920

[4] Kouanda A Watson R Binmoeller KF Nett A Hamerski C. EUS-guided gastroenterostomy for duodenal obstruction secondary to superior mesenteric artery syndrome. VideoGIE 2021;6(1):14–5. 33490746 10.1016/j.vgie.2020.09.008 PMC7806496

[5] Xu MM Dawod E Gaidhane M Tyberg A Kahaleh M. Reverse endoscopic ultrasound-guided gastrojejunostomy for the treatment of superior mesenteric artery syndrome: A new concept. Clin Endosc 2020;53(1):94–6. 31794656 10.5946/ce.2018.196 PMC7003011

[6] Bronswijk M Fransen L Vanella G Hiele M van der Merwe S. Successful treatment of superior mesenteric artery syndrome by endoscopic ultrasound-guided gastrojejunostomy. Endoscopy 2021;53(2):204–5. 32559775 10.1055/a-1190-3228

[7] Sobani ZA Rustagi T. Endoscopic ultrasound-guided gastrojejunostomy for the management of superior mesenteric artery syndrome. Am J Gastroenterol 2020;115(4):634–5. 32167937 10.14309/ajg.0000000000000567

[8] Welsch T Büchler MW Kienle P. Recalling superior mesenteric artery syndrome. Dig Surg 2007;24(3):149–56. 17476104 10.1159/000102097

[9] Sinagra E Raimondo D Albano D Superior mesenteric artery syndrome: Clinical, endoscopic, and radiological findings. Gastroenterol Res Pract 2018;2018:1937416. 30224915 10.1155/2018/1937416 PMC6129792

[10] ASGE Standards of Practice Committee, Storm AC Naveed M Fishman DS ASGE guideline on the role of endoscopy in the management of benign and malignant gastroduodenal obstruction. Gastrointest Endosc 2021;93(2):309–22.e4. 33168194 10.1016/j.gie.2020.07.063

[11] Chen YI James TW Agarwal A EUS-guided gastroenterostomy in management of benign gastric outlet obstruction. Endosc Int Open 2018;6(3):E363–8. 29527559 10.1055/s-0043-123468 PMC5842065

[12] Perez-Miranda M Tyberg A Poletto D EUS-Guided gastrojejunostomy versus laparoscopic gastrojejunostomy: An international collaborative study. J Clin Gastroenterol 2017;51(10):896–9. 28697151 10.1097/MCG.0000000000000887

